# Respiratory symptom patterns in healthcare workers in Germany: a prospective study during the SARS-CoV-2 pandemic

**DOI:** 10.3389/fpubh.2025.1717080

**Published:** 2026-01-09

**Authors:** Isabell Pink, Andrea Stölting, Anne Cossmann, Noemí Calderón Hampel, Luis Manthey, Marie Mikuteit, Sandra Steffens, Frank Müller, Georg M. N. Behrens, Alexandra Dopfer-Jablonka, Dominik Schröder, Christine Happle

**Affiliations:** 1 Department of Respiratory Medicine and Infectious Diseases, Hannover Medical School, Hannover, Germany; 2 German Center for Lung Research, DZL-BREATH, Hannover, Germany; 3 Department of Rheumatology and Immunology, Hannover Medical School, Hannover, Germany; 4 Department of Dermatology, Hannover Medical School, Hannover, Germany; 5 Dean’s Office, Curricular Development, Hannover Medical School, Hannover, Germany; 6 Department of General Practice, University Medical Center Göttingen, Göttingen, Germany; 7 Department of Family Medicine, Michigan State University, Grand Rapids, MI, United States; 8 German Center for Infection Research (DZIF), Partner Site Hannover-Braunschweig, Braunschweig, Germany; 9 Department of Pediatric Pneumology, Allergology, Neonatology, Hannover Medical School, Hannover, Germany

**Keywords:** COVID-19, healthcare workers, occupational risk factors, respiratory symptoms, SARS-CoV-2

## Abstract

**Background:**

Healthcare professionals (HCPs) faced unique exposure risks during the SARS-CoV-2 pandemic. This study investigated the prevalence and patterns of respiratory symptoms among HCPs, examining potential demographic, occupational, and vaccination-related risk factors during the early pandemic period.

**Methods:**

In this prospective observational study, 1,300 HCPs at a German academic medical center completed surveys about respiratory symptoms between March 2020 and April 2024. Participants reported five specific respiratory symptoms (cough, fever, dyspnea, sore throat, and impairment of taste/smell) and rated their intensity. These symptoms were grouped into one variable *acute respiratory tract symptoms* (ARS). Demographic and occupational data were collected at baseline. Symptom frequencies were compared with national surveillance data, and logistic regression analysis was used to identify potential risk factors.

**Results:**

Episodes of ARS demonstrated seasonality patterns similar to German national surveillance data. Female HCPs reported significantly more frequent and more severe rhinitis and sore throat symptoms, as well as more severe taste and smell impairment than male HCPs (*p* < 0.05). Participants with ARS were younger than those without. Despite an extremely low rate of laboratory-confirmed SARS-CoV-2 infections within the cohort during the study period (<1%), SARS-CoV-2 vaccination was associated with reduced risk for respiratory symptoms (OR 0.64, 95% CI 0.46–0.90).

**Conclusion:**

Our findings provide insights into respiratory symptom patterns during critical periods of a global pandemic and identify associations that merit further investigation. The protective association of SARS-CoV-2 vaccination with reduced respiratory symptom frequency, combined with seasonal patterns mirroring national trends, may inform occupational health strategies during future respiratory disease outbreaks.

## Introduction

The SARS-CoV-2 pandemic profoundly altered patterns of respiratory tract infections (RTIs) globally, with healthcare professionals (HCPs) facing unique exposure risks and challenges. During the early pandemic waves, HCPs experienced significantly higher infection rates compared to the general population (1) and suffered substantial physical and psychological consequences, including fear, anxiety, depression, and occupational stress (2).

Initial SARS-CoV-2 infections typically manifested with fever, cough, and dyspnea, alongside distinctive olfactory and taste disorders (3, 4). The clinical presentation evolved over time with the emergence of new viral variants and widespread vaccination uptake (5). Notably, the Omicron variant, which was predominant since January 2022, demonstrated reduced lower respiratory tract pathogenicity in experimental models, though tracheitis remained prevalent (6). McMahan et al. (7) observed lower pulmonary viral loads and less severe disease progression with Omicron compared to wild-type variants.

Vaccination has consistently proven effective in reducing COVID-19 severity (8, 9) and continues to provide protection against symptomatic infection, even with emerging variants (10). Recent evidence from the European ORCHESTRA consortium further strengthens the importance of vaccination and individual host factors in shaping the COVID-19 risk among healthcare workers: a large multicenter survey across four Italian university hospitals demonstrated that prior immunity and vaccination significantly modulated the risk for SARS-CoV-2 reinfection in healthcare professionals, highlighting the relevance of occupational exposure and immunological history (11). Additional data from this registry showed that body mass index, an important chronic health determinant, was associated with different risks of infection and distinct clinical patterns in pre- and post-vaccination periods (12). Together, these findings underscore how vaccination, host characteristics, and exposure environments jointly influence susceptibility to respiratory infections and should be considered when interpreting respiratory symptom trends in healthcare workers.

Several risk factors have been associated with severe COVID-19 outcomes, including male sex, cardiovascular comorbidities, and advanced age (13). While personal protective measures, particularly face masks, have demonstrated efficacy in reducing SARS-CoV-2 transmission (14). An increased risk of infections has been postulated for front-line healthcare workers, especially working on COVID-19 designated areas and predominantly working with COVID-19 patients (15, 16).

Understanding RTI patterns in HCPs during the pandemic is essential for developing targeted prevention strategies and occupational health protocols. We previously reported on the high levels of perceived risk in HCPs to become infected with SARS-CoV-2 during the early phases of the pandemic (17–19). However, limited data exist regarding the distribution of respiratory symptoms in healthcare settings during successive pandemic waves and how these patterns might have been influenced by vaccination status, imposed restrictions, or household composition.

This prospective observational study aims to characterize the prevalence and patterns of self-reported acute respiratory symptoms among HCPs during the COVID-19 pandemic. We examine these symptoms in relation to COVID-19 vaccination status, public health measures, and national respiratory disease surveillance data. Additionally, we investigate potential occupational and household risk factors that may have influenced acute respiratory tract symptom (ARS) occurrence in this high-exposure population during the early pandemic period.

## Methods

### Study design and participants

The CoCo study is an ongoing prospective observational cohort study registered with the German Clinical Trials Register (DRKS00021152, registration date: 7th April 2020) and approved by the Data Security Management and Institutional Review Board of Hannover Medical School (approval #8973_BO_K_2020). The study was conducted in accordance with the Declaration of Helsinki. Healthcare professionals (HCPs) working at Hannover Medical School, affiliated hospitals, or private practices in Lower Saxony, Germany, were enrolled in a dynamic cohort between April 2020 and April 2024. Inclusion criteria were: (1) working in the university hospital, (2) written informed consent, and (3) age ≥18 years. The study monitored respiratory infection symptoms. The whole protocol of the study has been published elsewhere (20).

### Data collection

Participants completed structured survey assessing their work situation and self-reported respiratory symptoms. During the initial 13 weeks of the study, surveys were conducted weekly, with participants reporting symptoms from the preceding week. Subsequently, the protocol was modified to a four-week surveying schedule, with participants reporting symptoms experienced during the previous weeks until January 2021. Subsequently, the administration of the survey occurred at more sporadic intervals. The questionnaires assessed five specific respiratory symptoms: cough, fever, rhinitis, sore throat, and impairment of taste or smell. Participants rated symptom intensity on a scale from 0 (no symptoms) to 10 (highest perceived severity) for the interval from the last to the present survey (20). Additionally, baseline information was collected, including: Pre-existing conditions and comorbidities (respiratory diseases, cardiovascular diseases, immunosuppression or others), SARS-CoV-2-vaccination status, workplace characteristics and home office arrangements, as well as household composition, including presence of children in the household.

### Outcome measures

The primary outcome was the prevalence and intensity of self-reported RTIs typical symptoms. For analytical purposes, individual respiratory symptoms were combined into a composite measure of ARS if one or more of the symptoms (cough, fever, rhinitis, sore throat or taste or smell impairment) were reported with at least a severity of 1 from a 0 to 10 scale.

### Statistical analysis

Normal distribution was assessed using the Anderson-Darling test. For categorical variables, absolute frequencies were calculated. For continuous variables, median and Quartile 1 and Quartile 3 (Q1, Q3; 25th and 75th percentile) were reported. Group differences were evaluated using the Wilcoxon rank sum test as all continuous variables were non-normally distributed and Pearson’s Chi-squared test for categorical variables. To account for multiple testing, the Bonferroni correction was used. A two-sided significance level of 5% was applied throughout. For bivariate analyses, all available data were used without imputation, and participants with missing values were excluded only from the respective comparison.

To assess the prevalence of the individual ARS over time, only timepoints with datasets in which at least 50 participants completed the survey, were included.

A comparative analysis was conducted on the baseline characteristics of all patients, examining differences between female and male participants. The occurrence of each respiratory symptom and the severity of the symptom burden were also compared between sexes regardless of the timepoint and number of completed surveys. The composite measures ARS was used to identify factors associated with the occurrence of acute respiratory symptom episodes. For this analysis participants were grouped in no symptoms (group no ARS) at any timepoint or at least one symptom at one timepoint (group ARS). A Bayesian logistic regression model was fitted using the brm() function from the brms package (21). Fixed effects included workplace, children at home, SARS-CoV-2-vaccination status, and sex. A random intercept was included for each individual (Pseudonym) to account for within-subject variability. This analysis was conducted using data without any missing values in the analyzed variables (workplace, children at home, SARS-CoV-2-vaccination status, sex). In order to investigate the direction of the effects of the predictors on the probability of respiratory symptoms, the Probability of Direction (pd) for each regression coefficient was calculated. When the value approaches 1, the probability of the observed direction is increased. In order to establish whether there were differences in the occurrence of ARS between the years under consideration, a comparison was made of the months February to April in the years 2020–2024. All statistical analyses and figures were generated using R Studio [Version 4.5.0 (2025-04-11), Posit Software, Boston, MA, USA].

## Results

### Study population and demographics

A total of 1,300 HCP were enrolled in this study and Table 1 shows the baseline characteristics. 446 persons reported to be nurses, 304 were medical doctors, 82 worked as medical assistants, and 302 in other occupations within the hospital (data not shown). 161 participants did not provide information about their specialization. Overall, 36 HCPs (19.0%) worked in outpatient settings, and 7 participants worked from home (3 completely, 1 mostly, and 3 sporadically). 30.1% of the participants reported comorbidities including respiratory diseases, cardiovascular diseases and, immunosuppression.

**Table 1:** Characteristics of HCP cohort.

Characteristic	Total (*N* = 1,300)	Male (*N* = 382)	Female (*N* = 917)	*p* value
Age				0.462^a^
Median (Q1, Q3)	40 (33, 52)	41 (35, 49)	40 (32, 53)	
Missing	17	3	14	
Respiratory symptoms				0.349^b^
No	430 (42.8%)	129 (45.1%)	301 (41.9%)	
Yes	575 (57.2%)	157 (54.9%)	418 (58.1%)	
Missing	294	96	198	
Workplace				0.920^b^
Emergency department	70 (37.0%)	22 (34.9%)	48 (38.1%)	
Intensive care unit	9 (4.8%)	3 (4.8%)	6 (4.8%)	
Normal ward	55 (29.1%)	19 (30.2%)	36 (28.6%)	
Outpatient clinic	36 (19.0%)	11 (17.5%)	25 (19.8%)	
Other	19 (10.1%)	8 (12.7%)	11 (8.7%)	
Missing	1,110	319	791	
Home office				0.577^b^
No	180 (96.3%)	60 (98.4%)	120 (95.2%)	
Children at home				**0.013** ^**b**^
Yes	58 (28.7%)	28 (39.4%)	30 (22.9%)	
Comorbidities				**0.005** ^**b**^
Yes	367 (30.1%)	87 (24.3%)	280 (32.4%)	
SARS-CoV-2 vaccination status				**<0.001** ^**b**^
No vaccination	316 (24.3%)	82 (21.5%)	234 (25.5%)	

Bold values are indicate statistically significant.

a Wilcoxon rank sum test.

b Pearson’s Chi-squared test.

### Sex differences

In addition to the baseline characteristics, Table 1 also demonstrates sex-specific differences in these characteristics. The cohort consisted of 70.5% (917) women and 29.4% (382) men, and 1 participant reported non-binary sex. The median age was 41 years for male and 40 for female participants. Male participants reported significantly more frequently to live with children in the household, and reported significantly higher rates of SARS-CoV-2 vaccination. Comorbidities were reported by 365 participants (28.1%), with female participants reporting significantly higher prevalence of comorbidities than male participants. 19.6% of participants reported one comorbidity, 2.9% two, and 0.5% three (data not shown). Respiratory diseases (6.8%) and cardiovascular diseases (5.8%) were the most prevalent comorbidities (data not shown).

**Figure 1:**
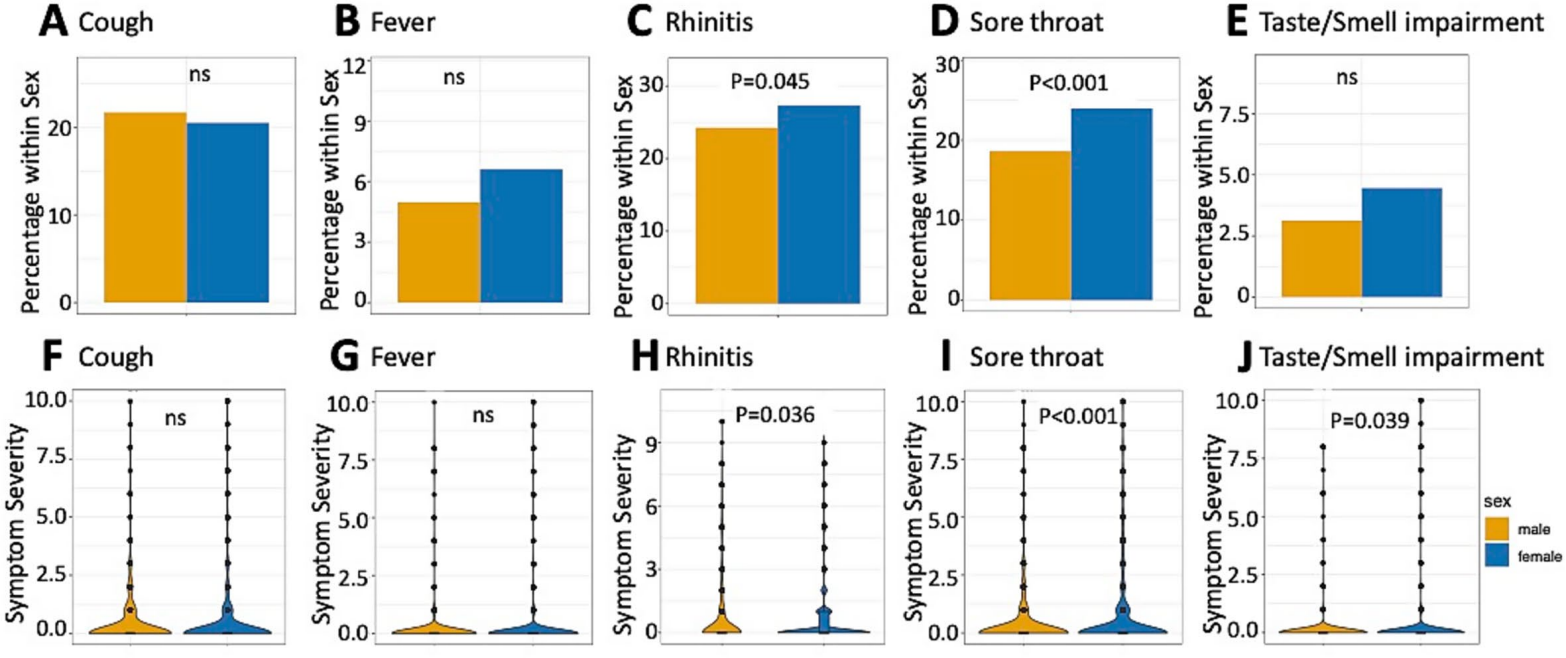
Sex-specific symptom reportings. **(A–E)** Bar plots of percentage of participants reporting the occurrence of symptoms within sex. **(F–J)** Violin plots of symptom severity between 0 (no symptom) and 10 (highest perceived intensity); ns, not significant.

Sex-specific analyses of symptom occurrence and severity are presented in Figure 1. A significant sex difference occurred with both increased frequency and severity of reported rhinitis and sore throat complaints, as well as more severe taste and smell impairments in female participants. No significant sex differences were observed for other respiratory symptoms.

**Figure 2:**
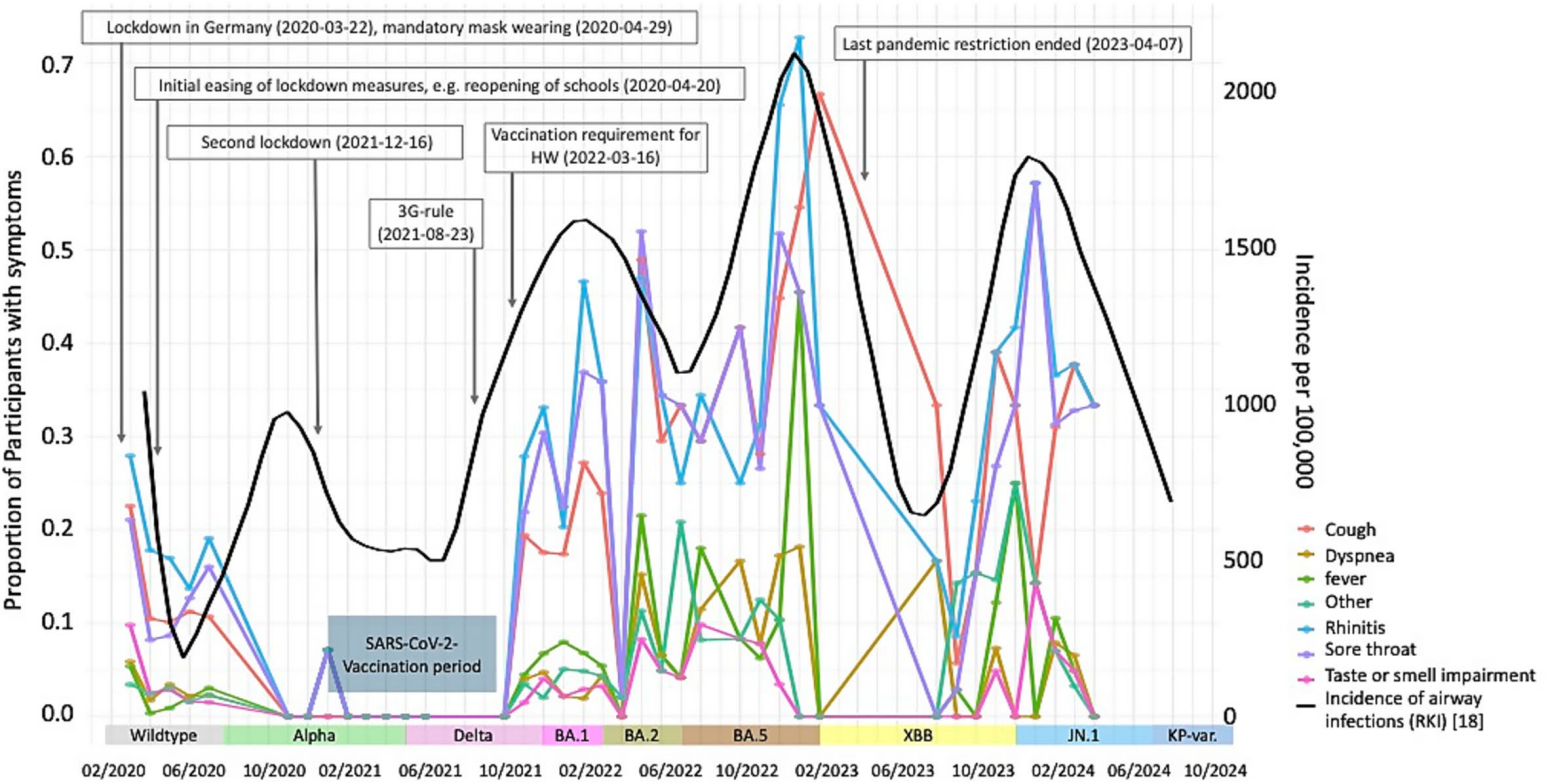
ARS reporting during the observational period. Lines denote fraction of participants reporting respective symptoms if symptoms were reported with a severity of at least 1. Black line represents the incidence data of airway infections of from the German national surveillance system (RKI) per 100,000 residents (22) (right scale). Measures implemented during the pandemic are modified from [18–20]; 3G-rule: ‘vaccinated, recovered, or tested’ policy; HW, Healthcare worker; KP-var., KP-variants including KP.2, KP.3, KP.3.1.1.

### Temporal trends in acute respiratory symptoms

Figure 2 illustrates the temporal distribution of ARS in the study cohort. For this analysis, only those timepoints were considered which contained datasets of a minimum of 50 completed surveys. Supplementary Table 1 contains details about the response rate for the various timepoints. For comparison, this graph also contains incidence data on airway infections from the German national surveillance system of the Robert Koch-Institute (RKI, black line in Figure 2) (22). Measures of infection control implemented during the pandemic and the different variants of SARS-CoV-2 are also indicated in Figure 2 (modified from (23–25)). Furthermore, the period of the initial SARS-CoV-2-vaccinations in our cohort is added. ARS typically associated with the “common cold” (rhinitis, cough, and sore throat) were reported more frequently than symptoms of systemic or severe lower respiratory tract infections, such as fever, dyspnea, and loss of smell/taste. The peaks of symptom prevalence in the cohort aligned with ARE-trends observed in national surveillance data (Figure 1). Seasonal peaks in the incidence of respiratory infections were observed. The initial peak occurred upon onset of the pandemic, followed by increases in ARS in autumn/winter 2021, winter 2022/2023, and winter 2023/2024. The first and second lockdown, which imposed severe restrictions on social interactions, resulted in a decline in symptoms and respiratory infections. In our cohort, SARS-CoV-2 vaccination were administered prior to the legal mandate requiring healthcare workers to be vaccinated (January till September 2021), which was shortly after begin of the second lockdown. Following the gradual easing of restrictions, a notable increase in respiratory symptoms was observed, even after the vaccination period. The courses of airway infections reported by the national surveillance clearly align with the occurrence of symptoms within the cohort. The last pandemic restrictions were lifted following the study period’s zenith of symptoms in April 2023.

When we analyzed the occurrence of ARS episodes during the different years of the pandemic, which is shown in Table 2. We found significant differences with the lowest rate in 2021.

**Table 2:** Rate of participants reporting ARS between February and April from 2020 to 2024.

Year	Rate (%)	95%-confidence interval (%)
2020	45.8	42.8–48.8
2021	18.2	2.3–51.8
2022	60.7	54.2–66.8
2023	33.3	0.8–90.1
2024	54.1	49.1–59.0

### Association between baseline characteristics and presence of ARS

**Figure 3:**
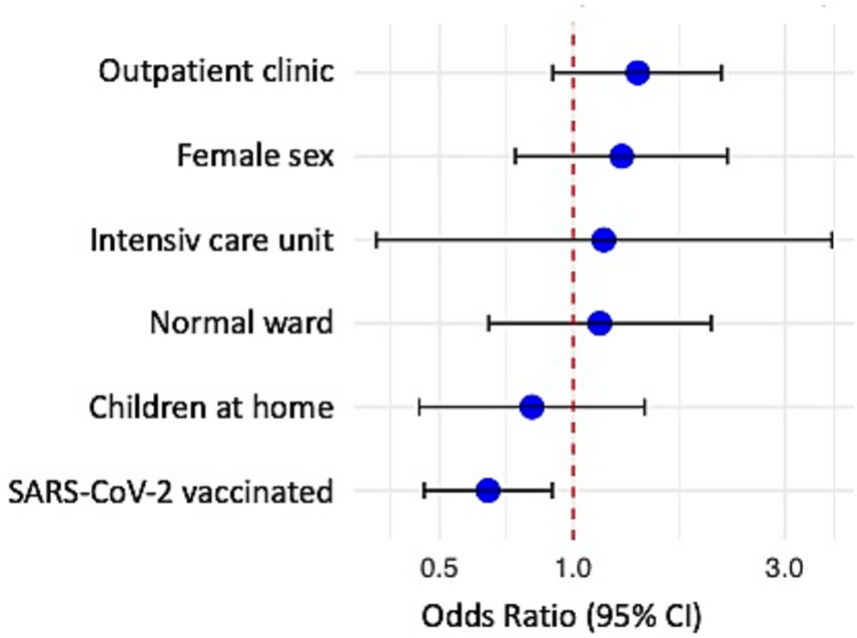
Forest ARS in association with HCPs occupational and social situation, as well as the vaccination status against SARS-CoV-2. Bayesian logistic regression was used to test for odds ratio; point symbolizes odds ratio, lines the 95% confidence interval (95% CI).

When comparing healthcare workers who reported any ARS episode vs. those who did not, we found a significant difference in the age: While participants with ARS had a median age of 41 years [Quartile 1 (Q1): 34, Quartile 3 (Q3): 55 years], those without had a median age of 43 years (Q1: 34, Q3: 55) (*p* = 0.023). Both groups consisted of a comparable proportion of Females (73% with ARS episode, 70% without ARS episodes). To analyzed the association of individual ARS episodes with demographic or occupational factors within our cohort, we performed a logistic regression analyses including 2,028 observations (*N* = 1,556 no ARS, *N* = 472 ARS) in 171 participants without any missing data of the variables throughout the entire study period (Figure 3). Logistical regression revealed that vaccination against SARS-CoV-2 at any time point of the observational period was associated with a significantly lower odds of ARS (OR 0.64, 95% CI 0.46–0.90; pd = 0.994).

## Discussion

In this prospective study of 1,300 healthcare professionals during the early phases of the SARS-CoV-2 pandemic, we observed that respiratory symptoms in HCPs largely followed seasonal patterns as indicated by national surveillance data. Furthermore, female HCPs experienced more frequent and severe rhinitis and sore throat episodes, and a reduced frequency of respiratory symptoms in HCPs vaccinated against SARS-CoV-2 was observed.

The temporal distribution of ARS in our cohort closely paralleled the trends observed in the German national surveillance data (22). During periods of strict non-pharmaceutical prevention strategies (NPS), we observed reduced symptom reporting, followed by increases that coincided with the gradual lifting of restrictions (26). This temporal correlation suggests that public health measures effectively impacted respiratory symptom patterns also in the healthcare settings with patient exposure. These insights are also relevant for occupational health practice, as the identified symptom patterns and associated risk factors may help refine targeted prevention strategies and infection-control protocols not only for SARS-CoV-2 but also for other respiratory pathogens circulating in healthcare settings. Analogues to our findings, an Italian study of 3760 HCP found considerable variations in the proportion of employees tested positive for SARS-CoV-2 and were symptomatic or asymptomatic across the different stages of the pandemic (27). Particularly low incidence was found at the beginning of the pandemic, comparable to our low incidence of ARE (see Figure 1). Higher proportions of infections were seen from November 2021 to June 2022 with a positive rate of around 37.0–38.0% (27). This is in line with our findings and underlines the influence of external factors such as different virus variants, vaccinations status, and social restrictions.

Our finding that female HCPs reported significantly more frequent and severe rhinitis and sore throat complaints, as well as more severe taste and smell impairment than their male counterparts, aligns with previous research showing sex-based differences in respiratory symptom perception and reporting (28). This observation may reflect both biological and psychosocial factors. Women have been reported to experience higher levels of pandemic-related anxiety and depression (29), which could potentially influence symptom perception and reporting behaviors. Additionally, biological mechanisms, including sex specific immune responses, may affect infection susceptibility and symptom manifestation (30). The predominance of women in our cohort (70%) reflects the higher proportion of female workers in healthcare settings in Germany and beyond (31). The observation regarding the younger age of participants reporting ARS is in line with the epidemiological date from the RKI, which report, that especially the German population aged 0–19 and 20–39 years showed a high incidence of COVID-19 in comparison to people aged 40–50 or >60 years (32). In addition, a higher risk of SARS-CoV-2-infections was also found in the above-mentioned HCP-cohort in Italy (27).

The observed association between SARS-CoV-2 vaccination and reduced frequency of respiratory symptoms may be explained by several mechanisms. A reduced risk for SARS-CoV-2-infections in HCP, especially after two doses of vaccine, has already been reported by Liviero et al. (27). SARS-CoV-2 vaccination may induce cross-immunity against other coronaviruses, which may also impact the occurrence of ARS (33, 34). Alternatively, psychological factors such as reduced vigilance toward symptoms following vaccination may have played a role. Particularly among people with high self-perceived risk for COVID-19 and severe disease courses, studies demonstrated considerable improvement in quality of life, mental health, and social participation after receiving a SARS-CoV-2 immunization (35–37). Moreover, a reduction in COVID-19 frequency may have played a role, but unfortunately, systematic pathogen surveillance was not performed throughout the study period.

Important limitations of our study include its single-center design, which may limit generalizability, and our reliance on self-reported symptoms without systematic pathogen testing. We were unable to correlate individual symptom trajectories with infection timepoints for SARS-CoV-2 or other respiratory pathogens. The questionnaire-based design carries inherent risks of subjective reporting bias and recall challenges. Some subgroup analyses were limited by small sample sizes, particularly for HCPs working from home. SARS-CoV-2-infections prior to the start of the study were not recorded. Although these are likely to be low due to the early start of the study, near to the beginning of the pandemic, this is a limitation worth mentioning. Moreover, we did not assess long-COVID, as a possibly relevant confounder, as the survey was conducted prior to the emergence of this disease. During the pandemic social mitigation strategies, individual behaviors and the different circumstances (e.g., wearing masks, exposure to respiratory viruses) and the changes of circulating virus variants which were not comprehensively addressed in our statistical calculations may have influenced ARE in our study populations. Symptom perceptions may has been influenced by mental health issues, which were also not systematically requested. Despite these limitations, our study provides valuable insights into respiratory symptom patterns among HCP during the COVID-19 pandemic period and identifies associations that merit further investigation.

## Conclusion

In conclusion, our findings demonstrate that during the pandemic, HCP experienced respiratory symptoms with seasonality patterns mirroring national trends. The observed age and sex differences in symptom reporting and the potential association between SARS-CoV-2 vaccination and reduced overall ARS episode risk highlight the complex interplay of biological, psychological, and occupational factors influencing HCP health during a pandemic. These insights may inform the development of targeted occupational health strategies for HCP in similar settings in the future. Further research should include comprehensive pathogen testing to better understand the specific etiologies underlying reported symptoms and explore potential mechanisms by which vaccination against one pathogen might influence broader respiratory health outcomes among HCP.

## Data Availability

The raw data supporting the conclusions of this article will be made available by the authors, without undue reservation.
