# Supplementary material for: Respiratory symptom patterns in healthcare workers in Germany: a prospective study during the SARS-CoV-2 pandemic
Source: Front Public Health. 2026 Jan 9;13:1717080. doi: 10.3389/fpubh.2025.1717080 (PMC12833979; doi:10.3389/fpubh.2025.1717080)
Supplement: Supplementary file 1 [file Data_Sheet_1.PDF]

Supplementary Table 1. Details of the response rate for the various timepoint of Figure 1

Time Point	Response rate
1	204
3	159
4	192
5	209
6	133
7	167
8	102
9	152
10	168
11	139
12	137
13	131
14	106
16	130
17	409
18	576
19	75
21	343
23	121
24	54
25	63
26	415
